# Integration of bioinformatics and imaging informatics for identifying rare *PSEN1* variants in Alzheimer’s disease

**DOI:** 10.1186/s12920-016-0190-9

**Published:** 2016-08-12

**Authors:** Kwangsik Nho, Emrin Horgusluoglu, Sungeun Kim, Shannon L. Risacher, Dokyoon Kim, Tatiana Foroud, Paul S. Aisen, Ronald C. Petersen, Clifford R. Jack, Leslie M. Shaw, John Q. Trojanowski, Michael W. Weiner, Robert C. Green, Arthur W. Toga, Andrew J. Saykin, ADNI

**Affiliations:** 1 Center for Neuroimaging, Department of Radiology and Imaging Sciences, Indiana University School of Medicine, Indianapolis, IN USA; 2 Department of Medical and Molecular Genetics, Indiana University School of Medicine, Indianapolis, IN USA; 3 Center for Computational Biology and Bioinformatics, Indiana University School of Medicine, Indianapolis, IN USA; 4 Department of Biochemistry and Molecular Biology, Pennsylvania State University, University Park, PA USA; 5 Department of Pathology and Laboratory Medicine, University of Pennsylvania School of Medicine, Philadelphia, PA USA; 6 Keck School of Medicine of USC, University of Southern California, Los Angeles, CA USA; 7 Department of Neurology, Mayo Clinic Minnesota, Rochester, MN USA; 8 Department of Radiology, Mayo Clinic Minnesota, Rochester, MN USA; 9 Departments of Radiology, Medicine, and Psychiatry, University of California-San Francisco, San Francisco, CA USA; 10 Department of Veterans Affairs Medical Center, San Francisco, CA USA; 11 Division of Genetics, Department of Medicine, Brigham and Women’s Hospital and Harvard Medical School, Boston, MA USA; 12 The Institute for Neuroimaging and Informatics and Laboratory of Neuro Imaging, Keck School of Medicine of USC, University of Southern California, Los Angeles, CA USA; 13 Indiana Alzheimer Disease Center, Indiana University School of Medicine, Indianapolis, IN USA

**Keywords:** Whole genome sequencing, Imaging genetics, Gene-based association of rare variants, *PSEN1*

## Abstract

**Background:**

Pathogenic mutations in *PSEN1* are known to cause familial early-onset Alzheimer’s disease (EOAD) but common variants in *PSEN1* have not been found to strongly influence late-onset AD (LOAD). The association of rare variants in *PSEN1* with LOAD-related endophenotypes has received little attention. In this study, we performed a rare variant association analysis of *PSEN1* with quantitative biomarkers of LOAD using whole genome sequencing (WGS) by integrating bioinformatics and imaging informatics.

**Methods:**

A WGS data set (*N* = 815) from the Alzheimer’s Disease Neuroimaging Initiative (ADNI) cohort was used in this analysis. 757 non-Hispanic Caucasian participants underwent WGS from a blood sample and high resolution T1-weighted structural MRI at baseline. An automated MRI analysis technique (FreeSurfer) was used to measure cortical thickness and volume of neuroanatomical structures. We assessed imaging and cerebrospinal fluid (CSF) biomarkers as LOAD-related quantitative endophenotypes. Single variant analyses were performed using PLINK and gene-based analyses of rare variants were performed using the optimal Sequence Kernel Association Test (SKAT-O).

**Results:**

A total of 839 rare variants (MAF < 1/√(2 N) = 0.0257) were found within a region of ±10 kb from *PSEN1*. Among them, six exonic (three non-synonymous) variants were observed. A single variant association analysis showed that the *PSEN1* p. E318G variant increases the risk of LOAD only in participants carrying *APOE* ε4 allele where individuals carrying the minor allele of this *PSEN1* risk variant have lower CSF Aβ_1–42_ and higher CSF tau. A gene-based analysis resulted in a significant association of rare but not common (MAF ≥ 0.0257) *PSEN1* variants with bilateral entorhinal cortical thickness.

**Conclusions:**

This is the first study to show that *PSEN1* rare variants collectively show a significant association with the brain atrophy in regions preferentially affected by LOAD, providing further support for a role of *PSEN1* in LOAD. The *PSEN1* p. E318G variant increases the risk of LOAD only in *APOE* ε4 carriers. Integrating bioinformatics with imaging informatics for identification of rare variants could help explain the missing heritability in LOAD.

## Background

Late-onset Alzheimer’s disease (LOAD) is a progressive neurodegenerative condition with no validated disease modifying treatment. With the heritability of LOAD estimated to be as high as 80 %, a better understanding of the genetic susceptibility factors of LOAD would advance strategies for early detection and treatment [1, 2]. Recent large-scale genome-wide association studies (GWAS) have identified and confirmed approximately twenty-one LOAD-associated genes in addition to *APOE*, whose ε4 allele is the best established and the most significant genetic risk factor [3]. While about 50 % of LOAD heritability is accounted for by all of the known LOAD susceptibility genes including *APOE*, a substantial proportion of the heritability for LOAD remains to be identified [1]. A growing body of evidence highlighting the role of rare variants has opened exciting avenues for discovering novel genetic factors to explain some of the missing heritability and facilitate a comprehensive understanding of LOAD.

Rapid advancement of next generation sequencing technologies has facilitated the search for genetic susceptibility factors that influence disease risk and become a key technique for detecting pathogenic variants in human diseases [4]. Our understanding of the impact of the genetic variation on human diseases has been greatly advanced using high-throughput sequencing [5]. Whole genome sequencing (WGS) has been used to obtain the most comprehensive genetic variation of an individual and perform detailed evaluation of all genetic variation [6]. Several sequencing-based association studies could identify functional risk variants with large effects on LOAD pathogenesis within *TREM2, ABCA7*, and *PLD3* genes [7–11].

Pathogenic mutations in *PSEN1* are known to cause familial early-onset Alzheimer’s disease (EOAD) but common variants in *PSEN1* have not been found to strongly influence LOAD [12]. Thus, the association of rare variants in *PSEN1* with LOAD-related endophenotypes has received little attention.

Accumulating evidence shows that common and rare risk variants are likely to co-exist at the same locus (known as pleomorphic risk loci) [13]. Deep re-sequencing-based association studies could identify functional risk variants within known susceptibility genes such as *ABCA7* [10]. In this study, we performed a rare variant association analysis of *PSEN1* with quantitative biomarkers of LOAD using WGS. Integration of bioinformatics and imaging informatics will provide a comprehensive and integrative approach to identifying a LOAD-specific genetic variation. In particular, imaging genetics combines neuroimaging such as MRI and PET with genetics for studying the influence of genetic variation on brain structure and function [14]. Quantitative endophenotypes increase detection power for rare variant association analysis and give additional information to interpret the association of variants by suggesting potential biological mechanisms by which the identified variants could lead to disease [14].

## Methods

### Subjects

All individuals included in these analyses were participants in the Alzheimer’s Disease Neuroimaging Initiative Phase 1 (ADNI-1) and its subsequent extensions (ADNI-GO/2). The initial phase (ADNI-1) was launched in 2003 to test whether serial magnetic resonance imaging (MRI), position emission tomography (PET), other biological markers, and clinical and neuropsychological assessment could be combined to measure the progression of MCI and early AD. The ADNI-1 participants were recruited from 59 sites across the U.S. and Canada and include approximately 200 cognitively normal older individuals (healthy controls (HC)), 400 patients diagnosed with MCI, and 200 patients diagnosed with early probable AD aged 55–90 years. ADNI-1 has been extended to its subsequent phases (ADNI-GO and ADNI-2) for follow-up for existing participants and additional new enrollments. Inclusion and exclusion criteria, clinical and neuroimaging protocols, and other information about ADNI have been published previously [15] and can be found at www.adni-info.org. Demographic information, raw scan data, *APOE* and whole genome sequencing data, neuropsychological test scores, and diagnostic information are available from the ADNI data repository (http://www.loni.usc.edu/ADNI/). Written informed consent was obtained at the time of enrollment for imaging and genetic sample collection and protocols of consent forms were approved by each participating sites’ Institutional Review Board (IRB).

### Whole genome sequencing (WGS) analysis

**Fig. 1:**
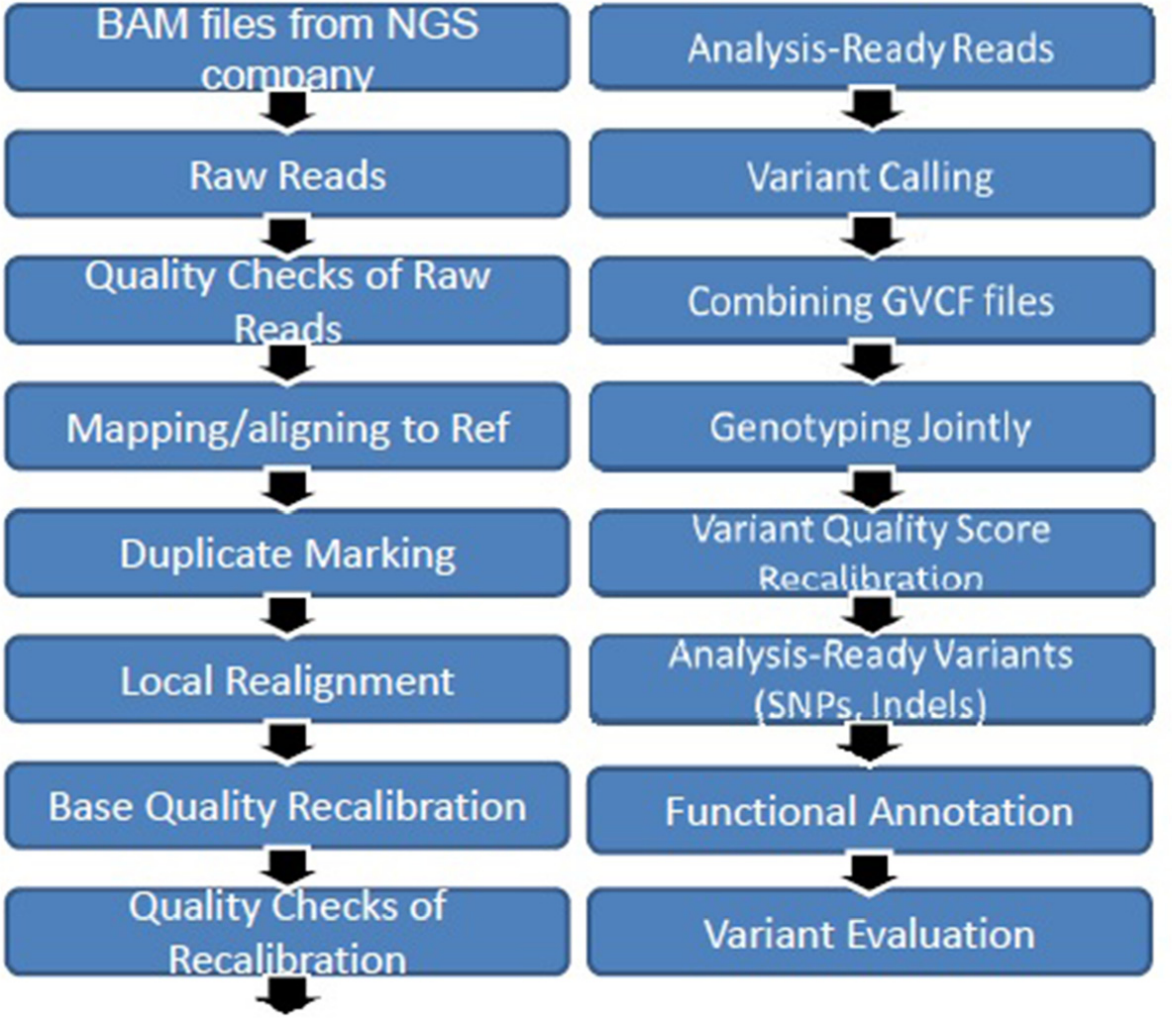
Pipeline for whole genome sequencing data analysis

WGS was performed on blood-derived genomic DNA samples obtained from 817 ADNI participants. Samples were sequenced on the Illumina HiSeq2000 using paired-end read chemistry and read lengths of 100 bp (www.illumina.com). The resulting Illumina qseq files were converted into fastq files, a text-based format for storing both sequence reads and their corresponding quality information in Phred format. Quality checks and read statistics are performed on raw sequence data in FASTQ format using FastQC. Short-read sequences are mapped to the NCBI reference human genome (build 37.72) using BWA, allowing for up to two mismatches in each read [16]. During the alignment, we use only bases with Phred Quality > 15 in each read to include soft clipping of low-quality bases, retain only uniquely mapped pair-end reads, and remove potential PCR duplicates. After completing initial alignment, the alignment is further refined by locally realigning any suspicious reads. The reported base calling quality scores obtained from the sequencer are re-calibrated to account for covariates of base errors such as sequencing technology and machine cycle [17]. Finally, the realigned reads are written to a BAM file for further analysis. The analysis-ready BAM files are analyzed to identify all variants with statistical evidence for an alternate allele present among samples using GATK HaplotypeCaller for multi-sample variant callings [17]. For variants which pass recommended variation quality criteria, ANNOVAR is used to annotate all variants (SNPs (single nucleotide polymorphism) and short insertion/deletions (indels)) [18]. We performed standard quality control procedures in WGS to assess the quality of WGS and to remove individuals and genetic variants with poor quality. We excluded variants that did not pass the variant quality score recalibration step using GATK in the WGS analysis pipeline and we removed variants whose genotype quality (GQ) scores < 20. The quality of the variant calls was assessed by comparing sequencing-derived SNPs with those obtained from the Illumina Omni 2.5 M genotyping array in order to estimate the concordance rate for each individual. Among 817 subjects, two subjects had concordance rates less than 99 % and had been removed from our analysis. The remaining subjects had a mean concordance rate of 99.9 % (Fig. 1).

### Subject selection

Since population stratification is known to cause spurious association in disease studies, we restricted our analyses to only subjects that clustered with CEU (Utah residents with Northern and Western European ancestry from the CEPH collection) + TSI (Toscani in Italia) populations using HapMap 3 genotype data and the multidimensional scaling (MDS) analysis (www.hapmap.org) [19].

### CSF measurements

Baseline CSF samples were obtained using previously reported methods for 3 CSF measurements (Amyloid-β 1–42 peptide (Aβ_1–41_), total tau (t-tau), and tau phosphorylated at the threonine 181 (p-tau_181p_)) as described [20]. Subjects who had at least one value greater or smaller than 4 SD (standard deviation) from the mean value of each of 3 CSF variables were removed from the analysis as extreme outliers [20].

### Imaging processing

T_1_-weighted brain MRI scans at baseline were acquired using a sagittal 3D MP-RAGE sequence following the ADNI MRI protocol [21]. As detailed in previous studies [22], a widely employed automated MRI analysis technique was used to process MRI scans: FreeSurfer V5.1 software (http://surfer.nmr.mgh.harvard.edu/). FreeSurfer was used to process and extract brain-wide target MRI imaging phenotypes (region volume and cortical thickness) by automated segmentation and parcellation. The cortical surface was reconstructed to measure thickness at each vertex on surface. The cortical thickness was calculated by taking the Euclidean distance between the grey/white boundary and the grey/cerebrospinal fluid (CSF) boundary at each vertex on surface. For surface-based comparison of the cortical thickness, all individual cortical surfaces were registered to a common surface template, which was an average created from all cognitively normal control subjects. The cortical thickness was smoothed with 10 mm FWHM Gaussian kernel to improve the signal-to-noise ratio and statistical power.

### Statistical analysis

Using all WGS-identified SNPs in the *PSEN1* gene region, we performed a gene-based analysis of rare variants with the optimal Sequence Kernel Association Test (SKAT-O) [23]. Rare variants were defined as variants with less than 1/√(2 N) = 0.0257 minor allele frequency (MAF) in our WGS sample. SKAT-O, which is an optimal unified approach for testing the association between rare variants and phenotypes in sequencing association studies and allows for heterogeneous effect of all variants within each gene, performed a score test for the model that includes the variants within each gene. Burden tests collapse information for multiple rare variants into a single genetic score and test for association of the score with a trait of interest [24]. In this study, we used the Morris and Zeggini (MZ) test as a burden test [25]. The MZ test uses a dominant genetic model to compute genetic scores as the number of rare variants for which an individual carries at least one copy of the minor allele [24]. The burden tests are powerful when a large proportion of variants are causal and effects are in the same direction. We performed association analysis using all SNPs. A single rare variant association analysis was tested using linear regression under a dominant genetic model in PLINK. Potential confounding factors (age, gender, years of education, intracranial volume (ICV), and MRI field strength) were used as covariates.

## Results

A total of 757 participants met criteria (quality controls and population stratification) for inclusion in analysis. There were 47 patients with Alzheimer’s disease (AD), 219 patients with early mild cognitive impairment (MCI), 232 patients with late MCI, and 259 cognitively normal older adults (HC) (Table 1). As expected, AD patients were found to have significantly lower Mini-Mental State Examination (MMSE) scores. Furthermore, the *APOE* ε4 allele frequency was significantly higher in patients with AD.

**Table 1:** Demographic characteristics of study participants

	HC	EMCI	LMCI	AD
N	259	219	232	47
Gender (M/F)	132/127	121/98	148/84	30/17
Age	74.3 (5.5)	71.1 (7.4)	73.2 (7.3)	75.2 (9.3)
Education	16.5 (2.7)	16.0 (2.7)	16.1 (3.0)	15.7 (2.7)
MMSE	29.1 (1.2)	28.4 (1.5)	27.5 (1.7)	22.9 (2.0)
*APOE* (ε4−/ε4+)	189/70	132/87	113/119	14/33

Values given are means (standard deviation), *MMSE* mini-mental state examination, *HC* cognitive normal older adult, *EMCI* early mild cognitive impairment (MCI), *LMCI* late MCI, *AD* Alzheimer’s disease

### Sequencing of the PSEN1 gene region

From an established WGS analysis pipeline, we found a total of 1,025 SNPs within a region of ±10 kb from *PSEN1*. Among 1,025 variants, there are 186 common (minor allele frequency (MAF) ≥ 0.0257) and 839 rare SNPs (MAF < 0.0257). Of 6 exonic rare variants, we found 3 nonsynonymous SNPs (Table 2).

**Table 2:** Single nucleotide polymorphisms (SNP) within *PSEN1* ± 10 kilobase (kb)

Common SNP (MAF ≥ 0.0257)	Novel	5	intronic: 4; downstream: 1
	Known	181	intergenic: 35; intronic: 141; 3′ UTR: 7; up (down) stream: 3
Rare SNP (MAF < 0.0257)	Novel	498	intergenic: 99; intronic: 372; exonic: 2; 3′ UTR: 15; 5′ UTR: 1; up (down) stream: 9
	Known	341	intergenic: 57; intronic: 257; exonic: 4; 3′UTR: 19; 5′UTR: 5; up (down) stream: 3

### Association of a PSEN1 rare variant p. E318G with CSF Biomarkers

To replicate the previous finding the risk variant (p. E318G) in *PSEN1* is significantly associated with LOAD-specific biomarkers, we performed an association using CSF biomarkers Aβ_1–42_ and t-tau, biomarkers of LOAD-associated pathologic changes in the brain. 583 participants with WGS data also had CSF biomarkers. A single variant association analysis showed that the *PSEN1* p. E318G variant was significantly associated with CSF biomarkers and increased the risk of LOAD in participants with *APOE* ε4 allele (*p* < 0.05; Table 3). Individuals carrying the minor allele of the *PSEN1* risk variant have lower CSF Aβ_1–42_ and higher CSF t-tau. However, the *PSEN1* p. E318G variant was not significantly associated with CSF biomarkers in participant group not carrying *APOE* ε4 allele. The percentage of participants without *APOE* ε4 allele having the minor allele (G) of *PSEN1* p. E318G is 6 % (21 participants), which is two times higher than that of participants with *APOE* ε4 allele (3 %; 7 participants) having the minor allele of *PSEN1* p. E318G. Furthermore, the *PSEN1* p. E318G variant was not significantly associated with CSF biomarkers in all participants regardless of the *APOE* ε4 status.

**Table 3:** Association results (*p*-values) of *PSEN1* p. E318G variant for quantitative trait analysis using a dominant model

	All participants (*N* = 583)	Participants with *APOE* ε4 (*N* = 234)	Participants without *APOE* ε4 (*N* = 349)
Aβ_1–42_	0.6199	0.0254	0.2733
t-tau	0.3795	0.0125	0.8673

### Gene-based association of PSEN1 rare variants with LOAD-specific imaging biomarkers

To test the hypothesis that *PSEN1* rare variants would be associated with structural changes in LOAD-related brain regions, we assessed entorhinal cortical thickness (EntCtx) as LOAD-related quantitative endophenotypes based on prior studies indicating that regional structural brain change in LOAD occurs initially and most severely in the entorhinal cortex and hippocampus before spreading throughout the entire brain [26]. Of the 755 of the 757 participants with WGS data, had usable high resolution T1-weighted structural MRI at baseline.

Gene-based association analysis of rare SNPs in the *PSEN1* gene with both SKAT-O and a burden test resulted that *PSEN1* rare variants were significantly associated with entorhinal cortical thickness after correction for multiple comparisons. The significant association was increased after adjustment for the *APOE* ε4 status (*p* < 0.05; Table 4).

**Table 4:** Gene-based association results (*p*-values) for imaging biomarkers (entorhinal cortical thickness (EntCtx)) using rare variants (MAF < 0.0257)

	Burden	SKAT-O	SKAT-O after adjusting for *APOE* ε4 status
Left EntCtx	0.009	0.015	0.010
Right EntCtx	0.027	0.046	0.032

**Fig. 2:**
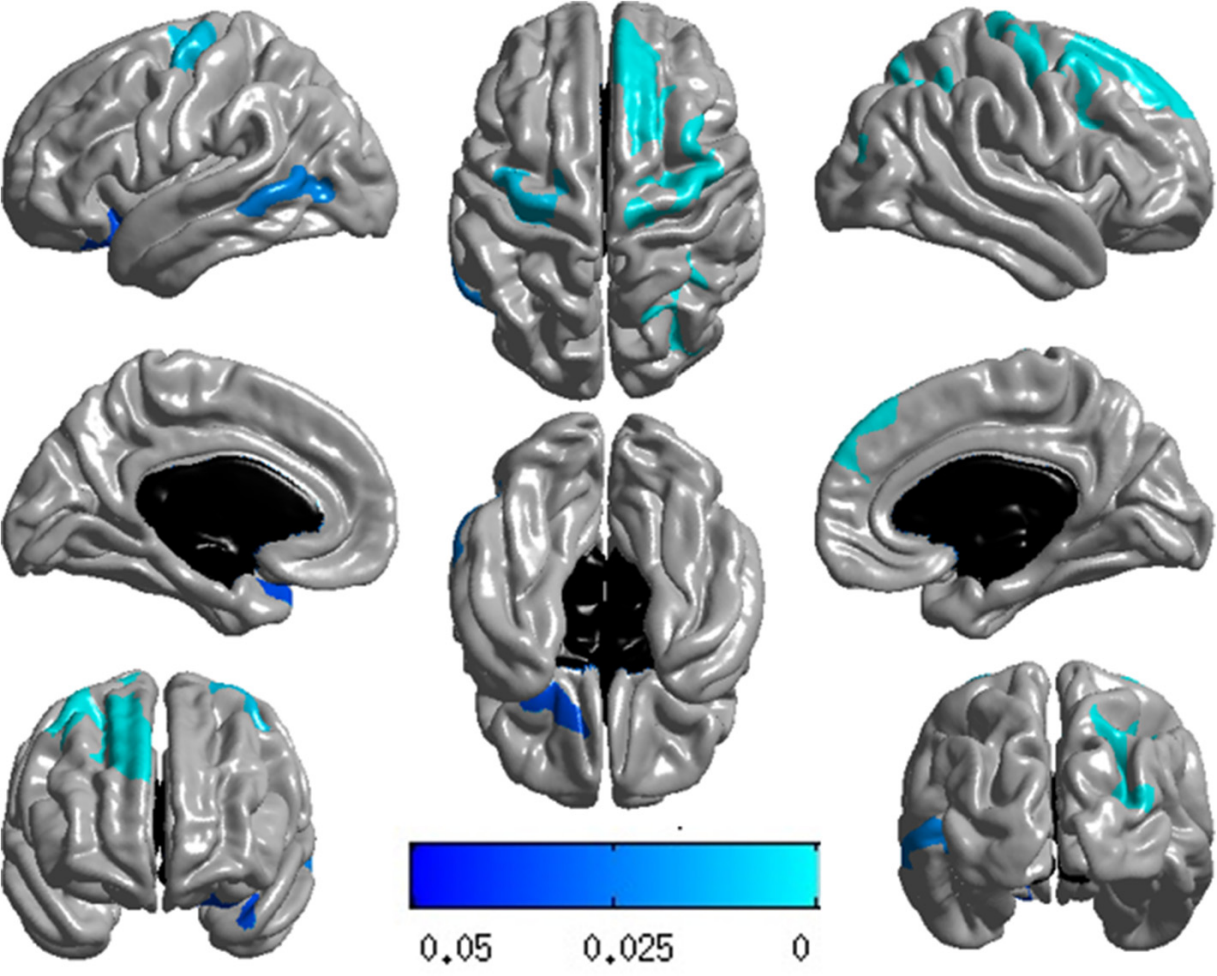
Surface-based whole-brain analysis results. A whole-brain multivariate analysis of cortical thickness was performed on a vertex-by-vertex basis to visualize the topography of genetic association in an unbiased manner. Statistical maps were thresholded using a random field theory adjustment to a corrected significance level of *p* = 0.05

To examine the cortical topography of all rare variants in *PSEN1*, an unbiased whole-brain multivariate analysis of cortical thickness was performed on a vertex-by-vertex basis to detect additional regions of association. We collapsed all rare variants into single risk score by combining minor allele counts into a single risk score with a dominant genetic model. A general linear model was constructed using age, gender, year of education, MRI field strength, and ICV as covariates. A random field theory based adjustment was used to correct for multiple comparisons to retain a 0.05 level of significance. Figure 2 displays the results of the main effect of all rare variants after adjusting for *APOE* ε4 status. Highly significant clusters associated with the risk score were found in bilateral temporal, bilateral parietal, and right frontal lobes regions (Table 5), where subjects having high risk scores showed thinner mean cortical thickness compared with the participants having lower risk scores (Fig. 2).

**Table 5:** Clusters significantly associated with polygenic risk scores of all rare variants within PSEN1 after multiple testing correction

Cluster	Cluster size (# of vertex)	Anatomic localization	Corrected *p*-value
1	3,798	right frontal lobe	8.96 × 10^−6^
2	4,247	right frontal lobe	2.29 × 10^−5^
3	3,436	right parietal lobe	1.23 × 10^−4^
4	2,718	left frontal lobe	7.11 × 10^−3^
5	1,915	left temporal lobe	2.04 × 10^−2^
6	2,078	left temporal lobe	3.33 × 10^−2^

### Association of common SNPs in the PSEN1 gene with LOAD-specific imaging biomarkers

A single variant and a gene-based association analyses of common SNPs in the *PSEN1* gene identified no significant association with entorhinal cortical thickness passed a multiple comparison correction (data not shown).

## Discussion and Conclusions

An association analysis of rare variants was performed on 757 ADNI WGS samples to investigate the influence of genetic variation in the *PSEN1* gene on LOAD-related imaging biomarkers. To our knowledge, this is the first study to show that *PSEN1* rare variants collectively show a significant association with the brain atrophy in regions preferentially affected by LOAD using integrated informatics methodologies. Our results indicated that rare variants in *PSEN1* were significantly associated with cortical thickness after correction for multiple comparisons and the significant association was increased after adjustment for the *APOE* ε4 status.

*PSEN1* regulates APP processing by affecting on gamma secretase enzyme which cleaves amyloid precursor protein (APP) and regulates amyloid-β accumulation which is a pathological hallmark of AD [27]. In addition, *PSEN1* plays an important role in Notch signaling pathway through the cleavage of the Notch receptor and Wnt signaling pathway [28, 29]. *PSEN1* locates on chromosome 14 and mutations in this gene are autosomal dominant and cause the early-onset AD [30]. Large-scale genome-wide association study (GWAS) showed that none of SNPs at the *PSEN1* locus reached to genome-wide significance [31]. Association studies investigating rare coding variants on the gene showed that rare variants in *PSEN1* were associated with sporadic LOAD [32, 33] and *PSEN1* p. E318G variant increased the risk of LOAD only in participants carrying *APOE* ε4 allele [12].

Performing advanced sequencing data analysis (bioinformatics) and human brain imaging analysis (imaging informatics) in an integrated approach enables us to identify blood-based biomarkers for risk or protection of LOAD, leading to an improved early diagnosis and prognosis, using LOAD-specific endophenotypes. Furthermore, use of quantitative endophenotypes substantially increase detection power for rare variant association analysis and holds great promise for discovery of variation mechanically related to AD pathophysiology. Confirmation of our results in independent and larger cohorts will be warranted.

In conclusion, we used whole genome sequencing to perform an association analysis of rare variants in *PSEN1* with LOAD-related imaging biomarkers. Our results illustrate the potential of integration of informatics methodologies to identify novel diagnostic/therapeutic targets for LOAD and understand the genetics and pathobiology of LOAD.
